# Dp-ucMGP as a Biomarker in Sarcopenia

**DOI:** 10.3390/nu14245400

**Published:** 2022-12-19

**Authors:** Natascha Schweighofer, Christoph W. Haudum, Olivia Trummer, Alice Lind, Ewald Kolesnik, Ines Mursic, Albrecht Schmidt, Daniel Scherr, Andreas Zirlik, Thomas R. Pieber, Nicolas Verheyen, Barbara Obermayer-Pietsch

**Affiliations:** 1 Department of Internal Medicine, Division of Endocrinology and Diabetology, Medical University of Graz, 8036 Graz, Austria; 2 CBmed, Center for Biomarker Research in Medicine, 8010 Graz, Austria; 3 Department of Internal Medicine, Division of Cardiology, Medical University of Graz, 8036 Graz, Austria

**Keywords:** dp-ucMGP, sarcopenia, biomarker, BioPersMed cohort

## Abstract

Sarcopenia is linked with an increased risk of falls, osteoporosis and mortality and is an increasing problem for healthcare systems. No satisfying biomarkers for sarcopenia diagnosis exist, connecting bone, fat and muscle. Matrix-GLA-protein (MGP) is an adipokine that regulates bone metabolism and is associated with decreased muscle strength. Associations of dp-ucMGP were analyzed in the BioPersMed cohort (58 ± 9 years), including 1022 asymptomatic subjects at moderate cardiovascular risk. Serum measurements of dp-ucMGP in 760 persons were performed with the InaKtif MGP Kit with the IDS-iSYS Multi-Discipline Automated System. DXA data (792 persons) measured with the Lunar iDXA system and physical performance data (786 persons) were available. Dp-ucMGP plasma levels correlate with sarcopenia parameters like gait speed (ρ = −0.192, *p* < 0.001), appendicular skeletal muscle mass (ρ = 0.102, *p* = 0.005) and appendicular skeletal muscle mass index (ρ = 0.112, *p* = 0.001). They are lower in persons with sarcopenia (*p* < 0.001) and higher in persons with reduced physical performance (*p* = 0.019). Persons in the lowest dp-ucMGP quartile have the highest risk for reduced muscle mass, decreasing with each quartile, whereas persons in the highest quartile have the highest risk of reduced muscle strength. Dp-ucMGP might be a good biomarker candidate in sarcopenia characterization.

## 1. Introduction

Sarcopenia is an increasing problem for healthcare systems since the number of people older than 60 years is expected to increase further in the future. In elderly individuals, sarcopenia leads to increased physical frailty and disability [1] and thus a loss of independence [2,3,4] and an increasing need for assistance during progression, often leading to long-term care placement [5]. Sarcopenia also contributes to mortality risk [6,7,8,9].

Sarcopenia is essentially a combination of a loss of muscle mass and function [8,10]. The onset of muscle mass loss is quite early: Healthy adults at the age of 40 years lose about 8% muscle mass per decade, which increases to 15% per decade past 70 years [11]. The impact of sarcopenia worsens with age, due to muscle strength decline, which shows an even more pronounced decrease due to changes in muscle quality [12].

The development of sarcopenia is mostly driven by three tissues: muscle, fat and bone. Bone and muscle interact directly along adjacent surfaces and via paracrine and endocrine factors. This interaction is mediated by fat, as several mechanistic crosslinks between bone and muscle are modulated by cells located in adipose tissue [13]. Matrix-GLA (γ-carboxyglutamate)-protein (MGP) might be a link connecting the three tissues since it is present and active in all of them. All three tissues express vitamin K-dependent gamma-glutamylcarboxylase, the enzyme responsible for the carboxylation of MGP during the vitamin K cycle. Carboxylated MGP is able to bind calcium and acts protectively in vascular calcification. In adipose tissue, MGP is highly secreted from adipocytes and acts as an adipokine [14]; in bone, it is expressed in osteoclasts and thus may play a role in bone metabolism [15,16]; and in muscle, lower vitamin K status is associated with decreased muscle and lower leg strength [17,18]. Furthermore, MGP might also influence muscle function via the preservation of the muscular microcirculation [19,20].

MGP is a small protein that can undergo two posttranslational modification steps, a vitamin-K dependent γ-glutamate carboxylation and serine phosphorylation [21], leading to the existence of four isoforms (phosphorylated-uncarboxylated (p-ucMGP), dephosphorylated-carboxylated (dp-cMGP), phosphorylated-carboxylated (p-cMGP), and dephosphorylated-uncarboxylated MGP (dp-ucMGP)) [22]. It is actually not clear, which isoforms are in general biologically active and what their systemic or tissue-specific functions might be. In the last 10 years, scientific approaches mostly focused on dp-ucMGP as an inactive form of MGP and surrogate parameter of vitamin K deficiency.

Since MGP is expressed and active in all three tissues mainly involved in sarcopenia development, it might be a good candidate as a biomarker in sarcopenia definition, especially since satisfactory molecules in this aspect are missing.

The aims of this work were to examine the role of dp-ucMGP as a potential biomarker in sarcopenia characterization by investigating its association with sarcopenia-related parameters and defining its influence on the risk to develop sarcopenia.

## 2. Materials and Methods

**Participants, study description and definition of comorbidities:** Data were generated in a population-based, single-center, prospective, observational study, the “Biomarkers of Personalized Medicine” cohort (BioPersMed), to evaluate novel biomarkers for the assessment of cardiovascular and metabolic disease and related complications [23]. The study cohort consists of asymptomatic subjects without manifest cardiovascular disease (CVD) but at least one classical risk factor for CVD defined by the European Society of Cardiology [1]. Community-dwelling adult persons (n = 1022) were recruited at the Divisions of Cardiology and Endocrinology and Diabetology at the Medical University of Graz. The study was approved by the institutional review board of the Medical University of Graz (EK-number 24-224 ex 11/12), and written informed consent was obtained from each participant. The BioPersMed study is conducted in compliance with Good Clinical Practice Guidelines Procedures (GCP) and complies with the Declaration of Helsinki and the Austrian laws. The design of the BioPersMed study, data management, biobanking and data analyses are compliant with the STROBE, STROBE-ME and STREGA recommendations.

**Figure 1:**
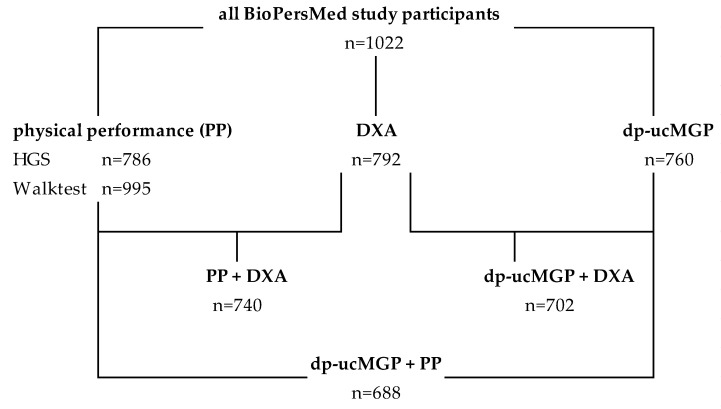
Flow chart of available measurements in the BioPersMed study participants: PP: physical performance; HGS: handgrip strength; DXA: energy X-ray absorptiometry; dp-ucMGP: dephosphorylated, uncarboxylated matrix-GLA-protein; n: number.

**Assessment of functional performance:** Muscle strength was determined by handgrip test in 786 individuals (see Figure 1). Handgrip strength (HGS) was measured with a hand-held Jamar hydraulic hand dynamometer according to the manufacturer (Patterson Medical Ltd., Huthwaite, Sutton-in-Ashfield, UK). The measurements were performed in triplicate for both hands and calculated as means for each hand in kilograms. For the definition of sarcopenia, the results of the weaker hand were used. We did not collect information about the dominant hand in this study. Physical performance was determined in 995 individuals by a modified 400 m walk test and chair stand test. The participants were asked to walk for 6 min as fast as possible and the distance in meters was measured. Gait speed was calculated for each participant: meters/second [m/s]: distance [m] divided by 360 [s]. Anthropometric measurements: body weight, precise to 0.1 kg, was determined using an electronic scale (model SECA 764, seca Deutschland, Germany); height, to 0.1 cm, was determined using a fixed stadiometer; waist circumference was measured in a standing position with a Lufkin tape positioned at the level of the umbilicus according to a MESA study [24,25]. The waist-to-height ratio was calculated by the division of waist circumference (cm) by height (cm). BMI was calculated as weight in kilograms divided by height in meters, squared (kg/m^2^). Study participants were allocated to three BMI classes (normal weight, overweight, obesity) according to the WHO (SuRF2 (who.int) [26]).

**DXA measurements:** Bone density and whole, as well as regional body composition, were measured in 792 individuals via dual-energy X-ray absorptiometry (DXA) with the Lunar iDXA system (GE Healthcare GmbH, Vienna, Austria) (see Figure 1). The least significant change of BMD (femur and lumbar spine) at 95% precision: root squared mean of 0.030/0.026 g/cm^2^, respectively, with a coefficient of variability (CV) of 0.024/0.032 g/cm^2^, respectively (2.43/3.24 percent), determined by the International Society For Clinical Densitometry’s advanced Precision Calculator tool (https://my.iscd.org/ accessed on 6 June 2022). The bone mass of extremities was calculated by the addition of the bone mass of arms and the bone mass of legs. The lean mass of arms and the lean mass of legs were measured by DXA.

**Definition of sarcopenia:** Cut-off values used in this work for the definition of sarcopenia are given in Table 1.

Sarcopenia was defined via HGS (handgrip strength), AMMI, ASM and gait speed.

Measurement of dp-ucMGP: Dp-ucMGP serum levels were measured in 760 persons (see Figure 1) using the InaKtif MGP Kit with the IDS-iSYS Multi-Discipline Automated System (both Immunodiagnostic Systems Holdings PLC, Tyne and Wear, UK). Dp-ucMGP quartiles were: Quartile 1 included the lowest plasma levels to 384 pmol/L, quartile 2: 385 to 462 pmol/L, quartile 3: 463 to 551 pmol/L and quartile 4: 552 pmol/L to the highest dp-ucMGP plasma level.

Data of sarcopenia-related parameters were available for 753 patients.

**Statistical analysis:** The nonparametric Mann–Whitney U test or Student’s *t*-test was used to compare quantitative variables between the groups, dependent on the absence or presence of normal distribution. Correlations between muscle, fat or bone parameters and dp-ucMGP were determined by using Spearman’s or Pearson correlation dependent on the normal distribution of data. The Chi-square test or Fisher exact probability test was used to compare categorical variables between groups and to test for linear trends across quartiles.

Binary logistic regression model analyses were performed. We used the presence of sarcopenia defined by AMMI, ASM or HGS as dependent variables in separate analyses, respectively. Dp-ucMGP quartiles (with quartile 1 as a reference) alone (unadjusted) or with age [years], alcohol consumption [drinks per week], smoking [pack years] and cardiorespiratory fitness (model 1) or additionally sex (model 2) as covariates were used. Since the number of covariates was limited due to only 80 cases of sarcopenia in total, we did not include sex (since the definition of sarcopenia is gender-specific) in our main analysis (model 1). To reduce the number of covariates, factor analysis was used to define cardiorespiratory fitness (extraction: maximum likelihood, based on Eigenwert > 1, varimax rotation, score calculated by regression). The parameters included were maximum oxygen uptake (VO_2_max) [mL/kg/min], anaerobic threshold [watt], maximum respiratory minute volume [l/min], maximum loading [watt] and maximum time of loading [sec] measured by ergospirometry (described in [1]).

Data are expressed as mean ± SD or percentages unless noted otherwise. A *p*-value < 0.05 was considered to indicate a significant difference for all performed analyses. SPSS Software version 26 (IBM Corp., New York, NY, USA) was used for all analysis.

## 3. Results

### 3.1. Description of Study Participants

Baseline data of sarcopenia-related parameters in all study participants with and without sarcopenia are given in Table 2. The sarcopenia-related parameters, HGS, gait speed and ASM, were selected as muscle proxies, fat mass, waist-to-height ratio and BMI to represent fat tissue and BMD and its normalized T-score as bone parameters.

As expected, the muscle parameters, HGS of the weaker hand, ASM and AMMI, differed significantly in persons deemed sarcopenic by AMMI. Additionally, the selected fat and bone parameters differed significantly between the two groups (see Table 2).

In total, 46 persons had decreased muscle strength measured by HGS; decreased muscle mass by AMMI was found in 126 persons and 102 persons by the use of ASM. No person showed a gait speed higher than 0.8 m/s. The definition of sarcopenia in 14 persons according to gait speed was only due to the non-completion of the task. No individuals were identified as sarcopenic by the stand up and walk test.

### 3.2. Dp-ucMGP Levels in Sarcopenia

**Figure 2:**
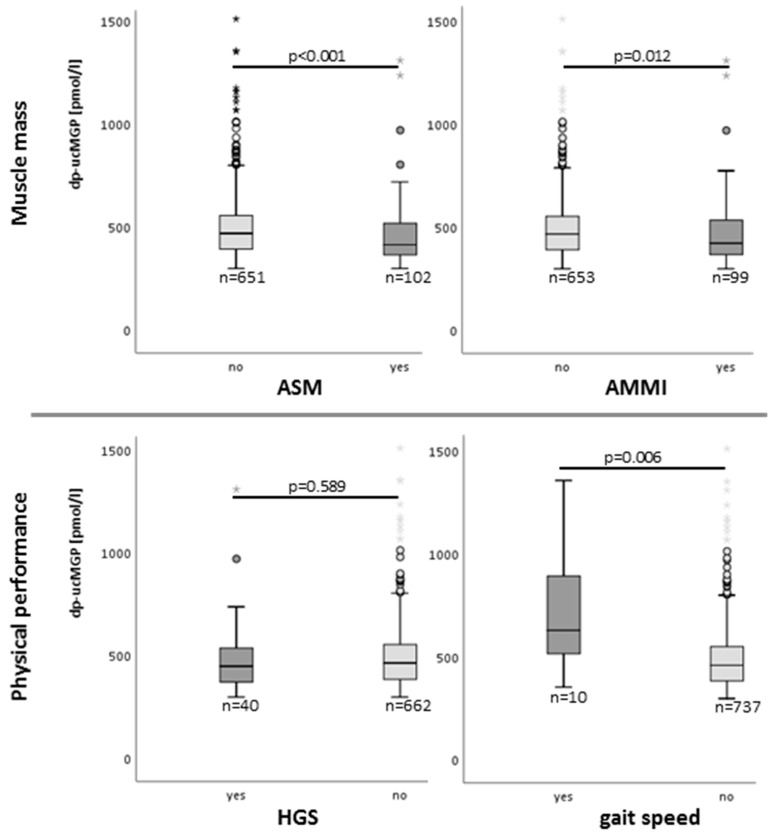
Dp-ucMGP levels in persons with and without reduced muscle mass or physical performance. Dark grey box plots represent the presence of decreased muscle mass or physical performance, whereas light grey boxes represent their absence. Dp-ucMGP: dephosphorylated, uncarboxylated Matrix-GLA-protein; pmol: picomol; L: liters, n: number; ASM: appendicular skeletal muscle mass; AMMI: appendicular skeletal muscle mass index; HGS: handgrip strength. Stars represent high extreme values and light or dark grey dots high potential outliers.

Dp-ucMGP levels in individuals with and without sarcopenia significantly differed regardless of whether gait speed, AMMI or ASM was used for sarcopenia definition (see Figure 2). The Dp-ucMGP levels were significantly higher in persons with reduced muscle function according to gait speed (716 +/*−* 306.6 vs. 491 +/*−* 164.4 pmol/L) and significantly lower in persons with reduced muscle mass according to AMMI (472 +/*−* 202.9 vs. 497 +/*−* 165 pmol/L) or ASM (449 +/*−* 183.2 vs. 499 +/*−* 167.5 pmol/L) compared to persons with normal muscle mass and function. Dp-ucMGP did not significantly differ between persons with and without reduced HGS (514 +/− 259 pmol/L and 493 +/− 163 pmol/L, respectively). For details, see Figure 2.

### 3.3. Dp-ucMGP Serum Levels and Sarcopenia-Related Parameters

**Figure 3:**
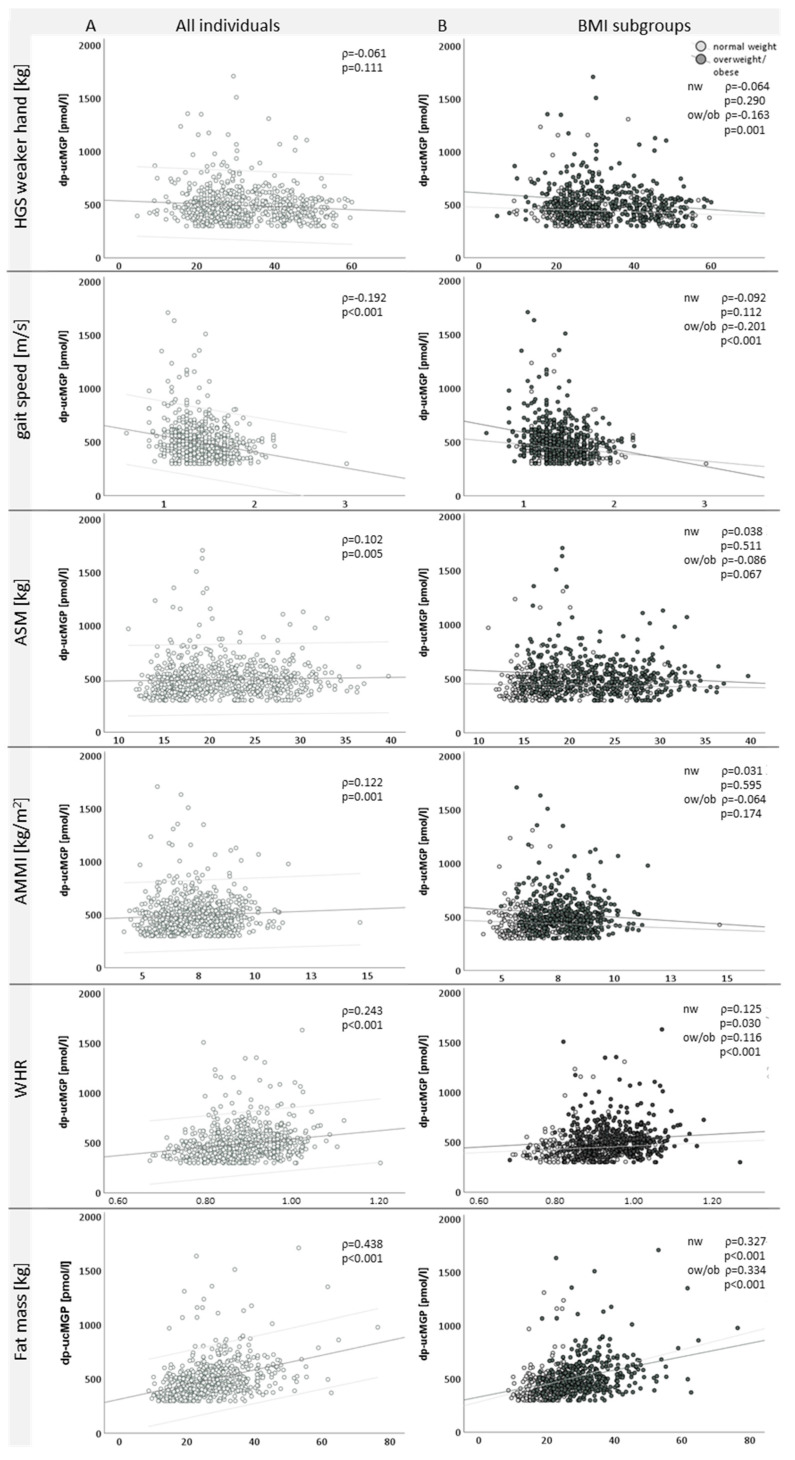
Circulating dp-ucMGP levels and correlations with sarcopenia-relevant parameters in all (**A**) and normal and overweight/obese (**B**) individuals. Correlation analysis between circulating dp-ucMGP and muscle parameters. Spearman’s rho (ρ) and *p* values are indicated. In all individuals, the 95% confidence interval is indicated by light grey lines. HGS: handgrip strength; ASM: appendicular skeletal muscle mass; AMMI: appendicular skeletal muscle mass index; WHR: waist-to-hip ratio; BMI: body mass index; kg: kilogram; min: minutes, L: liter; nw: normal weight; ow: overweight; ob: obese.

In all study participants, dp-ucMGP correlated positively with ASM, AMMI (see Figure 3), fat mass (ρ = 0.438, *p* < 0.001) and waist-to-height ratio (ρ = 0.405, *p* < 0.001) and negatively with gait speed (see Figure 3). No correlation was seen with the bone parameters investigated (BMD: Pearson correlation ρ = 0.013, *p* = 0.724), T-score: (Pearson correlation ρ = 0.015, *p* = 0.698). In sarcopenic individuals, according to AMMI, the dp-ucMGP levels correlated negatively with gait speed and positively with fat mass (Pearson correlation ρ = 0.226, *p* = 0.023) and waist-to-height ratio (Pearson correlation ρ = 0.289, *p* = 0.003).

As dp-ucMGP correlated positively with the parameters of fat distribution, we performed a subgroup analysis in all individuals with and without sarcopenia according to AMMI in normal and overweight/obese study participants (see Table 3).

In overweight/obese persons, dp-ucMGP correlated with HGS, gait speed and ASM negatively and positively with fat mass and the waist-to-height ratio (see Figure 3). In sarcopenic overweight persons, no correlations were seen with dp-ucMGP.

### 3.4. Analysis of Dp-ucMGP Quartiles in Sarcopenia

#### 3.4.1. Baseline Characteristics of the Subgroups

Details of the baseline characteristics of the study cohort subgrouped in dp-ucMGP quartiles are given in Table 4.

Chi-square analysis of all dp-ucMGP quartiles in individuals with and without sarcopenia defined by AMMI and ASM revealed a significant difference between the number of sarcopenic persons (*p* = 0.023 and *p* = 0.002, respectively). A linear-by-linear association (AMMI: 6.214; df = 1, *p* = 0.013 and ASM: 13.935; df = 1; *p* < 0.001) was found among all dp-ucMGP quartiles

#### 3.4.2. Dp-ucMGP and Sarcopenia Risk

**Figure 4:**
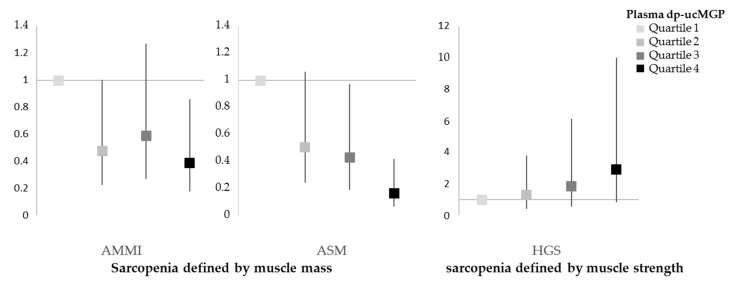
Odds ratios with 95% confidence intervals for prevalent sarcopenia features according to dp-ucMGP quartiles in 700 study participants. Adjustment: age [years], alcohol consumption [drinks per week], smoking [pack years] and cardiorespiratory fitness. Boxes with darkening shades of grey represent the dp-ucMGP quartiles.

We performed binary logistic regression analysis to determine the effect of dp-ucMGP (in quartiles) on the odds ratio for sarcopenia defined by AMMI, ASM or HGS. We used an unadjusted and two adjusted models: Model 1 was adjusted for age [years], alcohol consumption [drinks per week], smoking [pack years] and cardiorespiratory fitness; Model 2 included all parameters of model 1 and, in addition, sex. The odds ratios for each dp-ucMGP quartile on sarcopenia risk and sarcopenia features are given in Table 5 and Figure 4.

## 4. Discussion

The aims of this work were to examine the role of dp-ucMGP as a potential biomarker in sarcopenia characterization by investigating its association parameters of the three main tissues involved in sarcopenia development and with sarcopenia-related parameters and defining its influence on the risk to develop sarcopenia.

The main findings of the study were:

- Dp-ucMGP plasma levels correlate with the parameters of the three main tissues involved in sarcopenia development (muscle, fat and bone)
- Dp-ucMGP plasma levels correlate with sarcopenia parameters
  ○ gait speed, ASM and AMMI in all study participants
  ○ gait speed in sarcopenic participants
  ○ gait speed, HGS and ASM in all overweight/obese participants
  ○ gait speed in normal-weight sarcopenic participants
- Dp-ucMGP plasma levels are lower in persons with sarcopenia defined by AMMI and ASM in all persons and higher in persons with reduced physical performance.
- Persons in dp-ucMGP quartile 1 have the highest odds ratio for reduced muscle mass, decreasing with each quartile.
- Persons in dp-ucMGP quartile 4 tend to have the highest risk of reduced muscle strength

Muscle mass and muscle strength decline during aging, whereas the decline in muscle strength is higher than one would expect according to the decrease in muscle mass due to the loss of muscle quality [12]. The accumulation of fat in muscle is one of the mechanisms reducing muscle quality and is associated with lower muscle strength. Additionally, Baumgartner et al. showed that elderly persons with sarcopenic obesity had an elevated risk of becoming disabled (reviewed in [33]), further emphasizing the role of fat in the development of frailty. In this context, MGP might be interesting since in mice it is not only highly expressed in visceral fat but also contributes to adipogenesis and adiposity and is involved in adipocyte metabolism-like differentiation, the regulation of lipid storage and lipolysis [34]. MGP is also secreted from adipocytes but its actions as an adipokine request further investigation [14,35]. MGP is directly active in the skeletal muscle, where it regulates the expression of myogenic regulatory factors and myostatin [36]. In muscular microvasculature, MGP regulates angiogenesis and capillary function, as shown in animal models, which could additionally influence muscle mass and strength [37]. Muscle and bone are well connected since mechanical loading increases skeletal muscle mass, which in turn increases bone mineral content, bone mass and bone strength (reviewed in [38]). MGP might also influence the development of sarcopenia via bone since it seems to be important in bone metabolism. MGP is further potentially involved in the maintenance of bone health, since in mice, it promotes osteoblast proliferation, differentiation and mineralization, and in cell culture experiments, it inhibits osteoclast differentiation and bone resorption [39,40]. A further indication of MGP’s importance is a negative association of dp-ucMGP with BMD at the lumbar spine and a positive association with BMI [41]. The increased risk of reduced muscle strength in persons in the highest quartile of dp-ucMGP plasma levels might be explained by its relatively high positive correlation with fat mass. A high total fat mass might be a hint to increased fat incorporation in the muscle and declining muscle strength and quality. This effect seems to be vitamin K independent. The increased risk of muscle loss of persons in quartile 1 indicates the presence of a vitamin K-dependent action of MGP in muscle maintenance, where ucMGP would be inactive. Furthermore, the loss of muscle mass in these people is in line with the findings of Ahmad et al. [2], who demonstrated that the knockdown of MGP leads to the downregulation of myostatin expression (although also surprisingly of myotube formation). Chew and colleagues showed that high Myostatin serum levels correlate with frailty and low muscle mass in older adults [3]. MGP might thus be targeting the muscle on a structural basis too. Vitamin K was identified as possibly contributing to osteosarcopenic obesity development by Kelly and colleagues [42]. Since Van den Heuvel et al. proposed that high plasma levels of dp-ucMGP are a marker of vitamin K deficiency, it is used as a surrogate parameter for vitamin K [43]. Clinical studies on aging or frailty create evidence in favor of both findings: Higher plasma concentrations of vitamin K1 and lower dp-ucMGP levels were associated with better physical performance in community-dwelling older adults, and a higher dp-ucMGP level was associated with weaker handgrip strength, indicating a vitamin K dependent mechanism of action of MGP [17,18]. In our opinion, a reduction in its effects on vitamin K should be taken with care since it is not clear if dp-ucMGP is biologically active or not. It would be only valid for actions requiring MGP interactions with calcium since vitamin K is essential for the gamma-carboxylation of MGPs GLA residues and enables cMGP to bind calcium. It is not known whether cMGP plays a role in intramuscular calcium homeostasis [36], but the regulatory function of tissue or intracellular calcium homeostasis is proposed in other tissues [34], [44,45,46]. Furthermore, vitamin K might exert direct actions (as shown for menatetrenone (MK-4) [47]) or contribute to sarcopenia development through other vitamin K-dependent proteins. Some evidence for the distinct functions of dp-ucMGP and vitamin K in sarcopenia development are given by clinical studies since neither did vitamin K1 supplementation change the appendicular lean mass nor did menaquinone-7 supplementation increase physical performance or handgrip strength in older individuals (reviewed in [47]). Furthermore, in the longitudinal aging study Amsterdam, the decline of muscle function was not related to vitamin K status [17,48]. Our data indicate both mechanisms: they are in line with findings of van Ballegooijen et al. and Shea et al., showing an association of higher dp-ucMGP with decreased gait speed, which we additionally find in overweight/obese but not normal weight persons, and lower handgrip strength, although we only detected the latter in overweight/obese individuals. We also saw only an association by trend with the muscle mass parameter ASM in overweight/obese sarcopenic persons. In sarcopenic persons, especially normal-weight individuals, dp-ucMGP was negatively associated with gait speed. Interestingly, in our cohort, the persons in the lowest dp-ucMGP quartile showed the highest odds ratio for decreased muscle mass and for sarcopenia defined by AMMI.

For sarcopenia therapy, currently, no satisfying medication exists, so it is limited to resistance training and dietary strategies involving protein supplements. In this context, the addition of vitamin K and vitamin D have been proposed to have positive effects [49,50] which might be attributed partly to cMPG or ucMGP actions. Another important part of the dietary intervention is its anti-inflammatory components. MGP might act as an anti-inflammatory agent and thus muscle protective since it is expressed in leucocytes and is able to suppress the cytokine production of activated T cells [51,52]. MGP is able to exert further functions, although not well investigated yet, by interaction with binding partners as shown for BMP2, Fibronectin or Stat5 [53,54,55]. In total, the composition of the protein taken in, the sufficient supply of all vitamins as well as a good portion of antioxidant and anti-inflammatory nutrients might help to prevent sarcopenia and improve sarcopenia-related parameters such as muscle mass, strength and gait speed and might also be important in sarcopenia progression and the prevention of cognitive impairment [56,57].

The main limitation of this study is the lack of availability of direct measurements of vitamin K to distinguish clearly between the effects of low vitamin K levels and dp-ucMGP action. Another limitation is the relatively low mean age of the study participants leading to low numbers of persons with reduced muscle mass or function. In this study, we decided to define sarcopenia only via decreased muscle mass or hand-grip strength, due to the low number of persons with reduced physical performance, which made a definition of sarcopenia by combining both data impossible. Another limitation is the use of BMI to determine the presence of overweight or obesity since it has various limitations and does not well represent body composition [58]. We diagnosed sarcopenia mostly by AMMI, which represents only an approximation of the true muscle mass [8,48], although it might underestimate sarcopenia in overweight/obese individuals [59]. People identified as having reduced physical performance mostly did not finish the task rather than exceeding the limits of 0.8 m/s. Although the reason for test abortion was not noted, we tried to exclude reasons due to injury or cardiovascular complications by carefully examining the medical histories and the data generated in the study.

The strengths of the study are the extensive clinical characterization of the study participants, the comparison of associations in sarcopenic and non-sarcopenic persons and the determination of the association of dp-ucMGP with sarcopenia risk. The relatively low mean age is not only a limitation but also a strength, since the age range between 40 to 60 years is ideal to detect the beginning of sarcopenia development, facilitating a higher impact of therapeutic actions.

Further work is needed to determine which MGP forms are biologically active and to identify the vitamin K-dependent and -independent actions involved in sarcopenia development.

## 5. Conclusions

Several facts hint that MGP might be a promising biomarker candidate in sarcopenia. Dp-ucMGP plasma levels are associated with the parameters of the three main tissues involved in sarcopenia development, muscle, fat and bone. Additionally, its plasma levels correlate, although not very strongly, with sarcopenia-related parameters. Dp-ucMGP levels are significantly different in persons with reduced muscle mass and physical performance. Furthermore, dp-ucMGP is associated with the odds of reduced muscle mass.

Further studies with more sarcopenic persons and vitamin K measurements are needed to fully determine the usefulness of uc-dpMGP as a sarcopenia biomarker and to investigate whether other MGP isoforms might be possible biomarkers for sarcopenia characterization or definition.

## Figures and Tables

**Table 1:** Reference values for decreased muscle mass or function.

Definition of Sarcopenia					
	Unit	Calculation	Cut-Off Values		Reference
			males	females	
HGS	kg	direct measurement	<27	<16	[27]
AMMI	kg/m^2^	ASM/height^2^	<7	<5.5	[28]
ASM	kg	lean mass of arms and legs–bone mass of arms and legs	<20	<15	[29]
gait speed	m/s	direct measurement	≤0.8 m/s		[30]
					[31]
400 m walk test	min	direct measurement	non-completion or ≥6 min for completion		[32]

HGS: handgrip strength; AMMI: appendicular skeletal muscle mass index; ASM: appendicular skeletal muscle mass; m: meters; s: second; kg: kilograms; min: minutes.

**Table 2:** Sarcopenia-related parameters in all persons and persons with and without sarcopenia according to AMMI.

	All Individuals		Sarcopenia According to AMMI				
	n = 753		no	n = 651	yes	n = 102	
	Mean	SD	Mean	SD	Mean	SD	*p*-Value
age [years]	57.1	8.7	56.6	8.2	57.8	8.7	0.204
***Muscle parameters***							
HGS weaker hand [kg]	31.8	10.8	32.2	10.9	29.5	9.8	**0.019**
gait speed [min]	1.4	0.3	1.4	0.3	1.4	0.2	0.297
ASM [kg]	21.1	5.2	21.8	5.1	17.0	3.7	**<0.001**
AMMI [kg/m^2^]	7.3	1.3	7.5	1.2	5.8	0.8	**<0.001**
***Fat parameters***							
fat mass [kg]	26.6	9.3	27.5	9.4	20.7	5.4	**<0.001**
waist-to-height ratio	0.54	0.08	0.55	0.08	0.48	0.06	**<0.001**
BMI [kg/m^2^]	26.5	4.6	27.1	4.4	22.5	2.6	**<0.001**
***Bone parameters***							
BMD [mg/cm^3^]	1.19	0.13	1.20	0.13	1.15	0.13	**0.001**
T-score	0.61	1.13	0.67	1.12	0.23	1.10	**<0.001**

dp-ucMGP: dephosphorylated, uncarboxylated matrix-GLA-protein; HGS: handgrip strength; ASM: appendicular skeletal muscle mass; AMMI: appendicular skeletal muscle mass index; BMI: body mass index; BMD: bone mineral density; kg: kilogram; pmol: picomol; L: litre; min: minutes; m: meters; cm: centimeters; mg: milligrams; SD: standard deviation; n: number. Significant results are given in bold numbers.

**Table 3:** Correlations of dp-ucMGP levels with sarcopenia-related muscle parameters, fat and bone parameters according to BMI subgroup.

	All Individuals				Sarcopenia According to AMMI			
	Normal Weight		Overweight/Obese		Normal Weight		Overweight/Obese	
	CC	*p*-value	CC	*p*-value	CC	*p*-value	CC	*p*-value
Physical performance		n = 275		n = 423		n = 76		n = 18
HGS weaker hand [kg]	−0.064	0.290	**−0.163**	**0.001**	−0.113	0.331	−0.294	0.294
gait speed [min]	−0.092	0.112	**−0.201**	**<0.001**	**−0.230**	**0.040**	−0.319	0.170
Muscle mass		n = 298		n = 455		n = 82		n = 20
ASM [kg]	0.038	0.511	−0.086	0.067	0.069	0.540	−0.119	0.617
AMMI [kg/m^2^]	−0.031	0.595	−0.064	0.174	0.028	0.810	−0.031	0.897
Fat parameters		n = 281		n = 421		n = 80		n = 20
fat mass [kg]	**0.270**	**<0.001**	**0.334**	**<0.001**	**0.230**	**0.040**	−0.074	0.758
waist to height ratio	**0.223**	**<0.001**	**0.300**	**<0.001**	0.209	0.060	0.246	0.296
Bone parameters		n = 281		n = 421		n = 80		n = 20
BMD [mg/cm^3^]	**−0.122**	**0.041**	−0.048	0.327	−0.088	0.440	0.098	0.682
T-value	**−0.154**	**0.010**	0.004	0.934	−0.161	0.150	0.106	0.656

CC: correlation coefficient (either Spearman’s rho or Pearson coefficient); normal weight: BMI: body mass index < 25, overweight/obese: BMI ≥ 25; HGS: handgrip strength; ASM: appendicular skeletal muscle mass; AMMI: appendicular skeletal muscle mass index; BMD: bone mineral density; kg: kilogram; L: liter; min: minutes; m: meters; cm: centimeters; mg: milligrams; SD: standard deviation; n: number. Significant results are highlighted in bold script.

**Table 4:** Baseline characteristics of the study cohort by dp-ucMGP levels in quartiles.

	Dp-ucMGP Q1		Dp-ucMGP Q2		Dp-ucMGP Q3		Dp-ucMGP Q4		*p*-Value
*dp-ucMGP range [pmol/L]*	*lowest to 384*		*385 to 462*		*463 to 551*		*552 to highest*		
*n*	*190*		*190*		189		*191*		
dp-ucMGP [pmol/L]	345	±28	423	±22	502	±25	706	±200	
males	40%		44%		49%		44%		0.405
age [years]	54	±7	55	±7	57	±8	61	±9	<0.001
Waist-to-hip ratio	0.89	±0.09	0.92	±0.08	0.93	±0.08	0.94	±0.08	<0.001
BMI [kg/m^2^]	24.5	±4.0	25.8	±3.7	26.8	±4.5	28.6	±4.4	<0.001
alcohol consumption [drinks/week]	3	±5	4	±7	3	±5	3	±6	0.050
bone mass extremities [kg]	1.34	±0.33	1.38	±0.34	1.44	±0.34	1.39	±0.33	0.053
ASM [kg]	20.21	±5.24	21.19	±5.59	21.80	±5.42	21.53	±4.99	0.012
AMMI [kg/m^2^]	6.99	±1.32	7.28	±1.44	7.38	±1.35	7.45	±1.27	0.004
HGS weaker hand [kg]	33.07	±10.75	31.30	±11.32	32.91	±11.35	30.59	±9.64	0.226
Gait speed [m/s]	1.47	±0.25	1.46	±0.23	1.44	±0.25	1.33	±0.24	<0.001
Smoking [pack years]	8.1	±13.1	8.6	±13.5	6.5	±11.3	8.3	±14.2	0.317

dp-ucMGP: dephosphorylated, uncarboxylated matrix-GLA-protein; T: tertiles; BP: blood pressure; BMI: body mass index; ASM: appendicular skeletal muscle mass; AMMI: appendicular skeletal muscle mass index; HGS: handgrip strength; pmol: picomol; L: liter; mm: millimeter; Hg: mercury; kg: kilograms; m: meters; SD: standard deviation; n: number. Numbers are given as mean +/− SD until otherwise stated; %: percent.

**Table 5:** Odds ratios for the association of dp-ucMGP quartiles with sarcopenia defined by AMMI, ASM or HGS.

Dp-ucMGP Q1				Dp-ucMGP Q2					Dp-ucMGP Q3					Dp-ucMGP Q4				
		OR	*p*	OR	B-Value		CI	*p*	OR	B-Value		CI	*p*	OR	B-Value		CI	*p*
**AMMI**																		
unadjusted	1	**0.026**		**0.523**	**−0.648**	**0.296**	**0.925**	**0.026**	**0.496**	**−0.700**	**0.279**	**0.884**	**0.017**	**0.493**	**−0.706**	**0.277**	**0.878**	**0.026**
Model 1	1	0.083		0.476	−0.743	0.225	1.003	0.051	0.589	−0.530	0.272	1.272	0.178	**0.389**	**−0.944**	**0.176**	**0.860**	**0.020**
Model 2	1	**0.049**		**0.463**	**−0.770**	**0.217**	**0.986**	**0.046**	0.504	−0.685	0.229	1.109	0.088	**0.346**	**−1.062**	**0.154**	**0.779**	**0.010**
**ASM**																		
unadjusted	1	**0.003**		0.637	−0.450	0.369	1.101	0.106	**0.496**	**−0.700**	**0.279**	**0.884**	**0.017**	**0.316**	**−1.153**	**0.165**	**0.605**	**0.001**
Model 1	1	**0.002**		0.506	−0.681	0.241	1.063	0.072	**0.430**	**−0.845**	**0.191**	**0.969**	**0.042**	**0.164**	**−1.809**	**0.064**	**0.418**	**<0.001**
Model 2	1	**0.003**		0.511	−0.671	0.244	1.073	0.076	0.454	−0.790	0.199	1.034	0.060	**0.171**	**−1.768**	**0.067**	**0.436**	**<0.001**
**HGS**																		
unadjusted	1	0.795		1.084	0.081	0.465	2.257	0.851	1.325	0.282	0.544	3.230	0.536	1.536	0.429	0.612	3.855	0.360
Model 1	1	0.381		1.294	0.258	0.441	3.795	0.639	1.838	0.609	0.552	6.123	0.321	2.898	1.064	0.840	9.994	0.092
Model 2	1	0.205		1.330	0.285	0.444	3.990	0.611	2.458	0.899	0.714	8.458	0.154	**3.688**	**1.305**	**1.007**	**13.502**	**0.049**

Dp-ucMGP: dephosphorylated, uncarboxylated matrix-GLA-protein; OR: odds ratio, CI: 95% confidence intervals, *p*: *p*-value; AMMI: appendicular skeletal muscle mass index; ASM: appendicular skeletal muscle mass; HGS: handgrip strength; Q: quartile; Model 1 adjusted for age [years], alcohol consumption [drinks per week], smoking [pack years] and cardiorespiratory fitness; Model 2 adjusted as model 1 and, additionally, for sex. Nagelkerke R^2^ and Homer and Lemeshow Test p-values for the unadjusted model: 0.021/1 (AMMI), 0.035/1 (ASM) and 0.004/1 (HGS), respectively, for model 1: 0.11/0.018 (AMMI), 0.205/0.485 (ASM) and 0.085/0.269 (HGS), respectively, and for model 2: 0.134/0.245 (AMMI), 0.207/0.545 (ASM) and 0.139/0.292 (HGS), respectively. Significant results are highlighted in bold script.

## Data Availability

Restrictions apply to the availability of these data. Data are available upon request. To receive access, a proposal must be submitted to Univ.-Prof. Dr. Barbara Obermayer-Pietsch (barbara.obermayer@medunigraz.at). Additional information can be obtained via the Research Management of the Medical University of Graz (tanja.ball@medunigraz.at).
